# An assessment of the informativeness of clinical trials in digital mental health

**DOI:** 10.1038/s44184-025-00177-z

**Published:** 2025-12-17

**Authors:** Bridianne O’Dea, Sally Rooke, Eliza-Rose Gordon, Fergus L. Lyons, Bojana Vilus, Neelesh Paravastu, Philip J. Batterham

**Affiliations:** 1 Flinders University Institute for Mental Health and Wellbeing, College of Education, Psychology and Social Work, Flinders University, Bedford Park, SA Australia; 2 Black Dog Institute, University of New South Wales, Sydney, NSW Australia; 3 The Matilda Centre for Research in Mental Health and Substance Use, University of Sydney, Sydney, NSW Australia; 4 Centre for Mental Health Research, The Australian National University, Canberra, ACT Australia

**Keywords:** Clinical trials, Anxiety, Bipolar disorder, Depression, Psychosis, Schizophrenia

## Abstract

Clinical trials in digital mental health have grown rapidly, yet little research has examined their informativeness. This study assessed the proportions of recent trials that met indicators of informativeness and explored related factors. Using stratified sampling from five trial registries, we randomly selected 25% (*N* = 152) of recent trials for depression, anxiety, and psychosis in high-income and low- and middle-income countries. Each trial was evaluated against 17 established indicators. On average, trials met only half of these (*M* = 8.9, SD = 4.57, range 2–17). Just 5.3% (*n* = 8) met all indicators, with methodological criteria more often satisfied than those related to ethical, equitable, or open research practices. Informativeness did not differ by disorder or region but was higher where trial documentation and reporting were more accessible, with notable variation across registries. Findings highlight that many digital mental health trials may lack value for stakeholders, underscoring the need to prioritise informativeness and improve registry reporting.

## Introduction

Clinical trials in digital mental health have rapidly proliferated, with several hundred trials registered in the last five years alone. While clinical trials are an essential step in establishing a rigorous evidence base for the safety and efficacy of interventions, there is increasing evidence that many trials are uninformative, defined as failing to produce value for researchers, consumers, clinicians, and policy makers^1^. Indicators of trial informativeness have been developed to gauge whether the outcomes of clinical trials translate to viable use for research stakeholders^2^. It has been estimated that over 50% of participants in healthcare studies have enroled in uninformative trials and the cost of these trials exceeds GBP £726 million^3^. High-quality clinical trials are essential for generating reliable evidence, maintaining public trust, allocating resources wisely, and advancing scientific knowledge in health and medicine. Trials that lack informativeness may violate many of these principles and compromise the expectations of research participants, funders, and the broader scientific community^3^.

Increasingly, academics have attempted to describe and quantify the informativeness of clinical trials in health and medicine. Zarin and colleagues^2^ posited that informativeness was the ability of a trial to guide clinical, policy, or research decisions and was influenced by the four key domains of trial importance, design quality, feasibility, and analytical integrity. They argued that informative trials must (i) address an important and unresolved scientific, medical, or policy question; (ii) be designed to provide meaningful evidence related to this question; (iii) be demonstrably feasible; (iv) be conducted and analysed in a scientifically valid manner; and (v) report methods and results accurately, completely, and promptly. Guided by these conditions, Hutchinson and colleagues^1^ assessed the informativeness of clinical trials across three diseases (ischaemic heart disease, diabetes mellitus, and lung cancer) and found that only one in five trials demonstrated adequate feasibility, reporting, importance, and design. Notably, the proportion of informative trials did not differ between diseases, indicating shared challenges in design, conduct and reporting across fields of medicine. Furthermore, Hutchinson and colleagues^1^ found that the various criteria of informativeness were unmet at similar rates, indicating the need for attention across all stages of the trial lifecycle. Certain types of clinical trials were found to be at greater risk of compromised informativeness due to poor blinding, trial phase, and lack of an industry sponsor^4^. There is also evidence to suggest that the importance of informativeness indicators in clinical trials differ across research stakeholders^5^. In particular, the voices of research participants and consumers are not yet adequately represented in informativeness indicators despite many triallists using lived experience to guide trial design^6^. Furthermore, when developing consensus on informativeness indicators, Gelinas and colleagues^7^ argued that the challenges to informativeness vary by research type. Thus, investigating informativeness in all areas of health and medicine from the perspectives of several key stakeholders is essential for improving our understanding of the barriers and facilitators to ensuring research value.

Digital mental health is broadly defined as the field of knowledge and practice associated with the development and use of digital technologies to improve mental health. The landscape of digital mental health is rapidly evolving, with numerous clinical trials being conducted to evaluate the effectiveness of various digital interventions including but not limited to web-based programs, wearables, smart devices, and virtual reality, for improving individuals’ mental health. There are several reasons why clinical trials of digital mental health interventions (DMHIs) may require a specialised focus in relation to informativeness. Many clinical trials in digital mental health are decentralised and conducted outside of clinical settings, such that they are siteless, virtual, remote, and home-based. Furthermore, many participants in digital mental health trials are not patients within a healthcare system but users within the community. These participants often receive self-directed digital interventions with varying levels of human supervision and in-person contact with clinical trial teams. It has also been argued that many digital mental health trials are limited by slow and inadequate recruitment lacking in diversity, high failure rates, and the poor fit of interventions for health systems and individuals^8^. Although some trials successfully recruit participants, Hall and colleagues^9^ found that nearly two-thirds failed to reach their baseline sample targets, with even fewer achieving follow-up targets. Furthermore, digital mental health trials may further exacerbate inequities to mental healthcare given that many geographic locations with low densities of service providers also have reduced access to reliable and fast Internet^10^. Control treatment definitions and selection in digital mental health trials also present unique challenges, often requiring additional control elements including the receipt of sham technologies that can be costly to develop^11^. The lack of long-term follow-up data in digital mental health trials and the inconsistent handling of dropouts has also been argued to limit the informativeness of digital mental health trials for clinical practice^12^. There are also emerging ethical issues in digital mental health, such as data privacy and the capacity to ensure informed consent remotely, which may compromise the informativeness of clinical trials in this field^13,14^. Similarly, safety assessments within digital mental health trials have also been found to be lacking and worthy of specialised guidelines^15^. Together, these factors introduce unique considerations for trial design and conduct in digital mental health and thus, likely informativeness.

Reporting standards are also relatively new in digital mental health and there has not been a study on the concordance of digital mental health trials with the purpose-designed CONSORT E-Health checklist. While this standard was developed to improve the suboptimal reporting of digital mental health trials^16^, the CONSORT E-Health checklist is only applied when researchers are publishing outcomes of randomised controlled trials and only when journals require it. A recent review of CONSORT concordance for AI trials found that only 10 out of 65 RCTs explicitly reported its use and only 3 of the 52 relevant journals explicitly endorsed or mandated its use^17^. This suggests that digital mental health researchers’ concordance with reporting guidelines is likely to be low. There are also significant variations in the reporting of safety events in digital mental health trials, with only 1 in 4 trials found to report these in outcome papers^18^. As such, it is unlikely that reporting standards alone improve trial quality and informativeness in digital mental health^19^.

There have also been several studies and commentary on the limited uptake of DMHIs beyond clinical trials, with many trials failing to address the variety of implementation barriers encountered in real-world settings^20,21^. In addition, with the increased requirements for post-market regulation through national standards and accreditation systems, stakeholders have expressed that clinical trials in digital mental must extend beyond a sole focus on empirical effectiveness evidence to also address data safety, fidelity to clinical guidelines, adherence, and engagement and to include data from routine care^22^. Thus, relying on the standards and practices originally developed for clinical trials in pharmacotherapies or other areas of medicine may be inadequate or a hindrance for ensuring informative trials in digital mental health^18^.

The digital mental health research community may also perceive the value of informativeness efforts differently to other fields of health and medicine. We recently surveyed an expert panel of international researchers in digital mental health (*n* = 25) and found that only one third were highly familiar with the concept of informativeness^5^. When asked to define the concept, these researchers focussed primarily on factors related to the translational potential of the interventions tested (72%) and trial methodology (64%)^5^. In addition, 80% of the researchers did not believe that improving trial quality was an essential priority for the field of digital mental health^5^. Greater investigation of the factors that influence trial informativeness will also better our understanding of how researchers, funders, and institutions can promote practices that improve the value of clinical trials in digital mental health.

To our knowledge, there has not yet been a systematic investigation of the likely informativeness of clinical trials in digital mental health. Consistent with the findings of Hutchinson and colleagues^1^, it is likely that many recent clinical trials in digital mental health may not meet important indicators of informativeness. Our team previously developed indicators of informativeness through a consensus exercise with digital mental health researchers, adults with lived experience, implementors of digital mental health, and trial statisticians and methodologists^5^. The current study aimed to determine the proportion of recent clinical trials in digital mental health that met these indicators. We also aimed to examine the relationship between informativeness and trial features such as disorder focus (depression, anxiety, psychosis), region (high-income countries versus low- and middle-income countries), trial registry, trial start date, and number of available outputs. By attempting to systematically assess the informativeness of recent trials, we hoped to identify strengths and weaknesses in trial quality in the field of digital mental health. This information can then be used to guide researchers, funders, and institutions on key considerations and practices that are likely to improve the informativeness of clinical trials in digital mental health, thereby increasing the quality and impact of the associated interventions.

## Methods

### Trial selection and collection of trial information

**Fig. 1:**
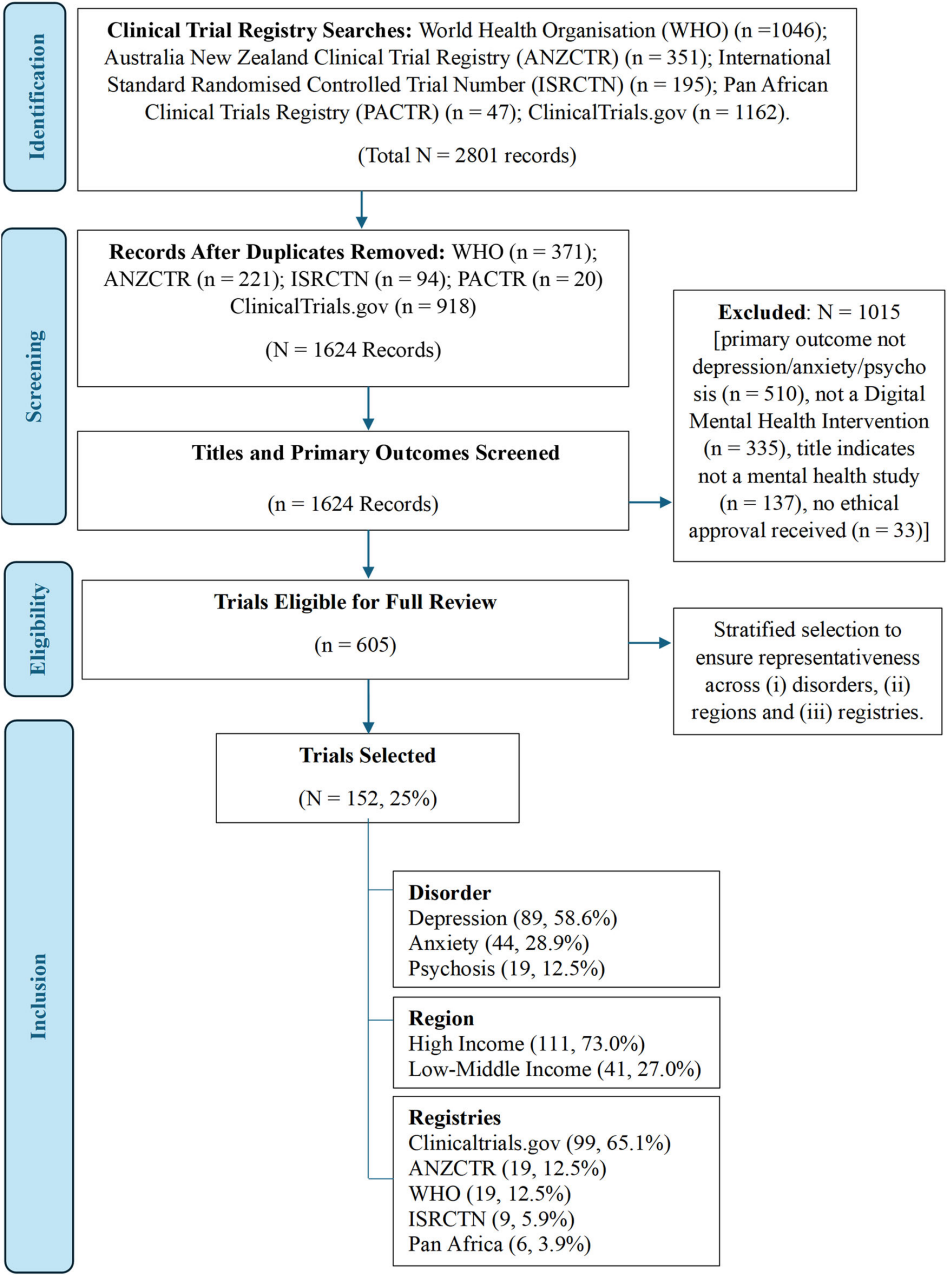
Digital mental health trial inclusions for the assessment of informativeness.

Our search flow and selection are outlined in Fig. 1. We first conducted a systematic search for recent (i.e., past five years) digital mental health trials in depression, anxiety, and psychosis registered on five trial registries (WHO, Clinical trials, ANZCTR, ISRCTN, Pan Africa) using the search method outlined in the Supplementary Material (S1). Our registries search followed published recommendations (Hunter et al.,^23^ Prang et al.^24^ with variations in search functionality across the registries accounted for. Three researchers piloted and validated the registry search results. After extracting trial information, removing duplicates, and screening for eligibility, we selected 25% (*n* = 152/605) for assessment using stratified random sampling with a web-based random number generator. The sampling was stratified by target primary disorder (i.e., depression, anxiety, psychosis), region, and registry to ensure representation, with psychosis trials and trials from low- and middle-income countries intentionally oversampled to ensure sufficiency. Source material for each trial was collated by two team members using a uniform search procedure. Source material included the trial registration entry (website-based entry and downloaded extracts) as well as any available protocols, documentation, or trial outcomes publications that were identified through Internet searches using the trial registration number. This also included any additional documentation provided on the trial registry, and/or in supplementary files from publications - most commonly statistical analysis plans, participant information statements, and consent forms. Authors were not contacted for trial information as the assessment deliberately relied on publicly accessible documentation to establish informativeness. Therefore, the assessment may have relied only on the trial registration information if no other source material could be found.

The start dates for the included trials ranged between 2nd of October 2017 and the 23rd of October 2023. We selected this date range to control for a time-effect whereby we intended to minimise confounding due to time (i.e. minimising the variability in time since a trial was started). The mean number of source material available for each trial was 1.9 (SD: 1.18, range: 1–6). Table 1 displays the source material available by year. Of the 152 trials that were assessed, 75 (49.3%) had only the registry entry information available. While 77 trials (50.7%) had more than one output available in the source material, the overall majority did not have a protocol (*n* = 110/152, 72.4%) or any outcomes papers (*n* = 104/152, 68.4%) available.

**Table 1:** Trial outputs by year of trial start date for the included trials (*N* = 152)

Year of trial start date	Number of trials started	Number of trials with registry entry only	Trials with protocols	Trials with one or more outcome papers	Trials with one or more supplementary outputs	Mean number of outputs in source material
2017	1	0	0	1	0	2.0
2018	3	2	1	0	1	1.7
2019	21	6	5	13	4	2.5
2020	25	8	10	12	4	2.2
2021	54	31	16	12	11	1.8
2022	43	25	8	10	14	1.6
2023	5	3	2	0	2	2.0
**Total**	**152**	**75**	**42**	**48**	**36**	**1.9**

### Assessment of informativeness

We conducted a standardised assessment of informativeness on all trials using a framework and matrix developed by the authors through a Delphi consensus study^5^, similar prior research^1^, and iterative pilot testing. The final instructions and assessment framework is included in the Supplementary Material (S2, S3). Two independent raters who were research staff with undergraduate or master’s qualifications in mental health research (selected from a pool of six trained raters) assessed the informativeness of each trial using the assessment matrix. The informativeness indicators were classified as “met” if there was any evidence that the trial had partially or fully addressed the described indicator using any of the information in the source material. We adopted a lenient approach, given that it was the first attempt to assess informativeness in the field of digital mental health. Raters marked indicators as ‘met’ in a standard Microsoft Excel template of the assessment matrix. Raters also inputted a hyperlink to the source material that was used to judge each the indicator. All raters received training on the assessment procedure, which included an instruction manual and video. The team of raters also met daily to discuss the indicator assessments and to resolve any questions. Where possible, disagreements were resolved by the initial raters through a discussion. A third independent rater (selected from a team of two discrepancy raters) conducted an additional assessment when consensus could not be achieved through discussion.

## Results

Table 2 presents the results of the informativeness assessment. On average, each trial met 8.9 of the 17 indicators (SD: 4.57). A total of 8 trials met all 17 indicators of informativeness (5.3%), and examples of these are provided in the Supplementary Material (S5).

**Table 2:** Percentage of trials assessed that met the endorsed indicators of informativeness and initial disagreement rates (*N* = 152)

Endorsed Indicator	Disagreement	Indicator met	
*Suitability of the team*	%	*n*	%
Any influence or involvement of industry, proprietary, commercial entities, or the creators of the DMHI being examined in the research have declared their conflicts of interest.	2.0	63	41.4
***Proposed methodology***	**%**	**n**	**%**
Specifies a clear and meaningful primary and secondary outcome(s) and endpoint(s) for the trial.	0.7	151	99.3
Provides clear information on how trial outcomes will be assessed at the respective endpoints.	3.3	151	99.3
Provides a sound justification for the selected DMHI, the comparators and/or control condition(s) with consideration of the likely efficacy, safety, and appropriateness for the intended population(s).	14.4	118	77.6
Measures the uptake and engagement with the DMHI throughout the trial.	19.0	104	68.4
The data management plan includes protocols to protect data integrity and reduce data loss.	26.8	77	50.7
The statistical analysis plan: (b) considers the impact of participant engagement in the DMHI on trial outcomes.	20.3	77	50.7
Includes clear instructions and expectations for use of the DMHI and/or comparators and controls examined in the trial.	28.1	83	54.6
Specifies how the safety of the DMHI will be monitored and assessed and the safety management procedures for participants using or exposed to DMHI in the trial.	15.7	65	42.8
The statistical analysis plan: (a) appropriately accounts for missing data.	6.5	60	39.5
***Ethical, equitable, and open research conduct***	**%**	**n**	**%**
Ensures the use of digital consent processes effectively meet the ethical standards for informed consent.	9.2	108	71.1
Addresses ethical issues related to emerging technologies and data collection within DMHIs including privacy, data security, confidentiality, and adherence to relevant local regulations.	15.7	68	44.7
Ensures equitable access and/or usability to the DMHI for the target population(s) and setting(s), including considerations of technology, language, literacy, and cultural appropriateness for the intended end-users.	20.3	51	33.6
Ensures timely and comprehensive reporting of results and outputs that will be accessible to stakeholders.	18.3	45	29.6
Monitors deviations to protocol and ensures the effects of these deviations will be managed.	12.4	34	22.4
Ensures any data monitoring procedures and follow-up actions are made clear to participants as part of informed consent.	19.0	25	16.4
***Potential and impact***	**%**	**n**	**%**
Provides a viable plan for translation of the DMHI post-trial, if found to be effective.	16.3	72	47.4

The highest levels of obtainment in the assessed trials were for the indicators related to the: primary and secondary outcome specification and measurement (99.3% of trials); justification for the selected digital mental health intervention (77.6% of trials); provision of information on digital consent (71.1% of trials); measurement of engagement with the digital mental health intervention (68.4% of trials); and provision of clear instructions for use of the digital mental health intervention (54.6% of trials). Only half of the trials met the indicators related to managing data loss (50.7%) and having a statistical analysis plan that accounted for participants’ lack of engagement with the intervention (50.7%). Less than half of all trials met the remaining indicators of informativeness. The lowest level of obtainment was for the indicators related to informing participants of any data monitoring procedures (16.4% of trials) and monitoring deviations to protocols (22.4% of trials). The total informativeness scores are summarised in Table 3 and the interquartile range (IQR) of 8 suggests high variability in informativeness across the selected trials.

**Table 3:** Overview of the variance in informativeness scores (*N* = 152 trials)

Statistic	Value
Possible range	0 to 17
Actual range of indicators met	2 to 17
Q1 (25th percentile)	5
Median interquartile range)	8 (5 to 13)
Mean (SD)	8.9 (4.57)

There was no significant difference in the mean informativeness scores between trials from low- and middle-income countries (*n* = 41, M: 9.1, SD: 4.39) and trials from high-income countries (*n* = 111, M: 8.8, SD: 4.66, *p* = 0.741). There were also no significant differences in the mean informativeness scores between depression (*n* = 89, M: 8.8, SD: 4.62), anxiety (*n* = 44, M: 8.2, SD: 4.35), and psychosis (*n* = 19, M: 10.8, SD: 4.55) trials (*p* = 0.110). There were significant differences in the mean informativeness scores across trial registries (*F* = 4.53, df = 4, 147, *p* < 0.05) with the highest mean informativeness score found for ISRCTN registered trials (M: 13.9, SD: 2.98) and the lowest mean score found for WHO registered trials (M: 7.9, SD: 4.57).

There was a significant relationship between informativeness score and the number of source materials available for each trial (*r* = 0.51, *p* < 0.001). There was a positive correlation between informativeness score and protocol availability (*r* = 0.41, *p* < 0.001) as well as informativeness score and trial outcomes papers (*r* = 0.32, *p* < 0.001). The informativeness of the registry-only trials varied (*n* = 75, M: 6.4, SD: 3.47, range: 2–17, IQR: 4.0) but were significantly less informative than the trials that had more than one piece of source material (*n* = 77, M: 11.6 SD: 4.22, range: 2–17, IQR: 7.0, *t* = −7.8, df = 145.9, *p* < 0.001). There was no significant correlation between trial start date and informativeness score (*r* = −0.11, *p* = 0.109). However, trial age was associated with the number of source materials (*r* = −0.21, *p* < 0.05), with higher mean outputs per trial in earlier years compared to trials that started in 2022 (+12.5% for 2021 and +37.5% for 2020).

### Inter-rater reliability

As shown in Table 2, there was some initial disagreement among the raters regarding whether many of the indicators were met or not. There was a total of 379 initial disagreements, with an average of 2.5 initial disagreements across the 17 indicators for each trial. The average rate of disagreement was 14.6% (κ = 0.85), which indicated moderate to substantial agreement. The indicators with the highest number of initial disagreements across trials were whether (i) the trial included clear instructions and expectations for use of the digital mental health intervention and/or comparators and controls examined in the trial (28.1% of trials recorded initial disagreement) and (ii) the data management plan included protocols to protect data integrity and reduce data loss (26.8% of trials recorded initial disagreement). Kappa values for each indicator are provided in Supplementary Material (S5).

## Discussion

This research aimed to assess the informativeness of recent clinical trials in digital mental health using indicators developed through consensus with key research stakeholders^5^. Based on information published in trial registries and materials retrievable through Internet searches, most of the assessed trials only met half of the indicators and only 1 in 20 trials met all. Indicators related to ethical, equitable, and open research conduct were less frequently met than indicators related to methodology. The methodological strengths of recent digital mental health trials included the specification of primary and secondary outcomes, the justification of the selected interventions, and the measurement of participant uptake and engagement. However, compliance with these aspects should be expected given these design features are relevant to almost all clinical trials of health interventions. The areas of improvement for digital mental health trials related to important participant-related factors including safety management, the equity and accessibility of interventions examined, the consideration of digital ethics, and dissemination of findings for all stakeholders. Furthermore, many of the indicators required for thorough interpretation of trial results, intervention safety, and replication studies such as statistical parameters, participant instructions, and safety monitoring were not met by most trials. Consistent with the findings of Hutchinson and colleagues^1^, the challenges to informativeness in this subset of digital mental health trials were similar across disorders and regions. Taken together, our findings suggest that many digital mental health trials may not be producing value for research stakeholders.

While clinical trials are essential for establishing the safety profile of digital interventions in the pre-market phase, only 40% of the assessed trials were found to adequately address safety management. The lack of transparent reporting in safety management is consistent with the findings of Taher and colleagues^15^ and suggests that a significant portion of digital mental health trials may have posed unmanaged risks to participants. Poor reporting of safety management also limits the capacity for methodological improvements in future trials by restricting researchers’ access to and knowledge of important safety considerations. Future work may benefit from upskilling researchers’ knowledge and use of new guidelines in digital safety management to improve the process of safety assessment of DMHIs in clinical trials^15^. In addition, mandating the publication of safety management protocols and participant information sheets in trial registries will also help to improve participants’ and researchers’ understanding and knowledge of safety management in past and current trials. Furthermore, while there have been increased calls for assurance of ethical conduct within DMHIs^25^, our evaluation found that few researchers appropriately declared competing interests. As proprietary knowledge and commercial interests become increasingly influential in the field of digital mental health, the integrity and credibility of digital mental health research may be questioned if the field does not improve its consideration and disclosure of these interests. The field may benefit from expanding digital mental health researchers’ understanding and acknowledgement of competing interests from “monetary or funding support” to include the “provision or creation of technology” and other material support invested by research, not-for-profit, and other for-profit organisations and in the creation and ownership of digital mental health technologies.

Few of the assessed trials appeared to have considered factors related to the equity and accessibility of DMHIs. While some trials provided participants with technology access (e.g., mobile phone or Internet), the majority required participants to have access to the Internet that was not funded by the research. As such, many digital mental health trials may be evaluating interventions that are not accessible to or effective for many demographic groups. Digital inequity is a central ethical issue for clinical trials in digital mental health as individuals with lower technical literacy and/or less access to the Internet are subjected to “digital exclusion,” thereby having no access to the benefits of effective online interventions^26,27^. Torous and colleagues^28^ asserted that digital exclusion is the single highest priority for achieving the full potential of digital health, and its inadequate consideration is likely to contribute to the widening of disparities in mental health treatment access and outcomes globally. There is increasing evidence that intentional, culturally appropriate, multipronged recruitment and retention strategies are effective for ensuring diverse participant inclusion in many areas of health research^29–31^. However, there is limited evidence that such approaches were adopted in the subset of the assessed trials. For example, most trials in high-income countries excluded non-English speakers. Given this, research funders may benefit from broadening the concept and assessment of scientific merit within clinical trial proposals to prioritise the equity and accessibility of interventions in diverse and marginalised populations. Enhancing data quality through diverse participant inclusion will also help to improve the informativeness of clinical trials in digital mental health. Furthermore, the limited attention paid to post-trial activity, including dissemination and implementation into practice, is also of concern, with fewer than 50% of trials providing any information on dissemination strategies. Progress in the DMHI field is likely to be accelerated with well-articulated plans for how findings might be used in practice, policy or future implementation of DMHIs. The need for greater consideration of dissemination applies both to trials that test new or established interventions and trials that address scientific questions (e.g., dismantling trials to understanding which elements of an intervention might be most efficacious), both of which may be informative to different stakeholders. However, our findings for this indicator may have been limited by the prospective nature of our assessment and the recency of many of the included trials. More detailed information on dissemination strategies may be provided at a later stage and continued follow-up of these trials would determine this.

The findings confirm the importance of transparent reporting and documentation in assessing and ensuring trial informativeness. Consistent with prior work^24,32^, our findings suggest that the formats of some trial registries fail to prioritise or elicit the information needed to assess the informativeness of clinical trials. Notably, trials registered on ISRCTN were found to have significantly higher informativeness scores than trials on some other registries, although noting only 9 of the trials were from this registry. This finding is likely due to the differences in reporting fields across registries. For example, the ISRCTN registry has a greater emphasis on informativeness-centric fields (e.g. ‘dissemination of research findings’) whereas the WHO registry provides only the minimum standards for trial registries and typically includes a hyperlink to the trial’s external country-only trial registry. Our findings strongly endorse the need for greater harmonisation and standardisation across trial registries to encourage a greater focus on informativeness-centric indicators and dissemination within academic and non-academic communities. Given methodology-related indicators were commonly met, digital mental health trials would benefit from additional input fields in trial registries to adequately capture informativeness criteria that are specifically relevant to this field.

Trials with more available source materials demonstrated higher levels of informativeness; however, on average, fewer than two pieces of publicly accessible material were available per trial. While delays in peer review may limit the timely publication of outcome papers, many trials had still not released other key study materials nor had they provided updates or disseminated results (e.g., participant summaries or pre-prints), despite being registered for up to five years. These findings reinforce concerns that many registries and trial teams are not committed to maintaining transparent, current reporting of trial progress and outputs across the trial lifecycle, which is an issue that becomes especially critical when journal publications are delayed. Moreover, even when source materials were available, there were substantial inconsistences in how information relevant to informativeness was reported. As the field of digital mental health rapidly develops, protocol and outcomes reporting standards will need to evolve^28^. For example, the CONSORT E-Health statement was designed only for randomised controlled trials and was last updated over 10 years ago. Many of the indicators initially excluded from the statement were among the indicators that were endorsed as essential to informativeness in our consensus research^5^. This divergence suggests that the CONSORT E-Health statement may benefit from an updated review to reflect changes in expectations within the digital mental health field. Funders and research institutions can support standardised reporting throughout the lifecycle of clinical trials in digital mental health by embedding them into proposal assessments, protocols, and trial monitoring reports, as well as including requirements for outcome reporting. Furthermore, funders’ open science and open access policies could be expanded to mandate the timely publication of both protocols and outcome papers in journals that adhere to appropriate reporting standards and as pre-prints, to improve the timeliness of reporting. Planning for data and material at the research design stage would enhance the long-term scientific value of trials, improve timeliness of reporting, facilitate cumulative progress in digital mental health research, and align with evolving standards for transparency and reproducibility.

Given that interrogating informativeness relies on accessible information, it was unsurprising to find that trials with higher levels of documentation were more likely to score higher on informativeness. As such, we cannot state with certainty that the low scoring trials failed to address these indicators at all, only that this information was not readily available. Some of the trials were also likely to have been impacted by a recency effect for outcomes papers, particularly for trials that started since 2022, as the trials may have still been underway. However, it would be reasonable to expect that trial registries had substantial information given that all trials had commenced at least six months before the assessment. This further confirms that trial registries are currently underserving both the academic and non-academic communities in the level and type of detail that they provide. Insufficient transparency in trial reporting (in registries and subsequent outputs) likely contributes to research waste by limiting opportunities for other investigators to build on or learn from ongoing studies. Comprehensive documentation is also essential for examining active ingredients of DMHIs, identifying core components, and understanding underlying mechanisms. Moreover, high-quality individual patient data meta-analyses rely entirely on researchers providing well-documented data and materials established from the outset. Many researchers now use a range of open science platforms to share trial materials but poor interoperability for record-keeping between platforms can contribute to further research waste. Future work on informativeness should therefore also consider the role of open science practices across the trial lifecycle, as these were not included in our current framework but are essential for research synthesis.

While the results also showed no significant differences in the informativeness scores between regions, the trials in low- and middle-income countries were often led by researchers from high-income countries. As such, our findings do not necessarily infer that researchers in low- and middle-income countries have access to the resources, infrastructure, training or support necessary to facilitate informative trials in digital mental health. It is also not clear from this research what factors drive researchers’ decision-making in clinical trials in digital mental health. Prior work has found that a lack of obligation, time, competent support, and financial resources impede some researchers’ selection and implementation of scientific trial designs and conduct^33^. Future research may benefit from using qualitative interviews to explore in more depth how trialists’ develop and design protocols, who is influential to their decision-making and what feedback and standards trialists value. Such research would help to identify the targets of influence and other agents of change for improving informativeness. Many researchers have argued for the need for greater efforts in lived experience engagement in research to ensure the translation and longevity of DMHIs^26,34,35^. Future work should focus on establishing methods for using lived experience priorities for informativeness to guide clinical trials in digital mental health.

Lastly, our use of a lenient dichotomous scale in the assessment framework may have inflated informativeness scores and obscured important variability between trials. Furthermore, while the assessors reflected that inter-rater reliability in informativeness scores was more consistent when using a dichotomous rating, disagreements still arose, largely due to poor reporting and ambiguity in the information provided by trialists. More in-depth workshopping and piloting of the assessment framework with other key stakeholders may further validate our assessment approach. Our decision not to extend the search window reflected a trade-off: while including more recent trials would have increased the sample size, it would not have changed how informativeness was defined or assessed and could have introduced bias by comparing trials at different stages of maturity. Future work could follow this trial cohort over time to monitor changes in informativeness as the more recent trials progress and report results. Future research could also extend this methodology to other mental and physical health conditions and examine differences across age groups, including child-focused trials.

This work represents a vital step in establishing and operationalising the concept of informativeness in digital mental health research. The results of our assessment confirm the need for a greater focus on enhancing the informativeness of clinical trials in this field. Key priorities include addressing informativeness factors related to equitable, accessible, and open research conduct as well as safety and post-trial dissemination. Future work should examine practical solutions to these problems that may include: (i) greater integration of informativeness-centric language and concepts in clinical trial funding and proposal assessments, (ii) introducing specialised post-funding scientific protocol reviews similar to those used in industry-funded pharmaceutical trials^1,4^, (iii) adopting a maturity model that enables informativeness to be examined across the lifespan of a trial^36^, and (iv) improvements in the reporting of trial information across all outputs including trial registries, protocols and outcomes papers, and at all stages of the trial lifecycle. Prioritising and evaluating the impact on these initiatives will further our understanding of how researchers, funders, and institutes can maximise the value of clinical trials within and beyond the scientific community.

## Supplementary information

Supplementary Information

## Data Availability

Data used in this study can be obtained by contacting the chief investigator by email: bridianne.odea@flinders.edu.au.
