# Supplementary material for: An assessment of the informativeness of clinical trials in digital mental health
Source: Npj Ment Health Res. 2025 Dec 17;4:66. doi: 10.1038/s44184-025-00177-z (PMC12711929; doi:10.1038/s44184-025-00177-z)
Supplement: Supplementary file 1 — Supplementary Information [file 44184_2025_177_MOESM1_ESM.pdf]

# Supplementary Material

## *S1. Selection criteria for digital mental health trials included in the assessment of informativeness*

| **Supplementary Table S1. Selection criteria for digital mental health trials included in the assessment of informativeness** | |
|---|---|
| Date range<br>*Search option on trial registers | Trials with a start date between 01/01/2019 and 01/01/2023 |
| Language | English |
| Registries - included | ANZCTR<br>Clinicaltrials.gov<br>ISRCTN<br>Pan Africa<br>WHO |
| Trial status | Active – recruiting OR<br>Active – not yet/no longer recruiting OR<br>Completed<br>AND<br>Has ethics approval |
| Population – Inclusion | Adults and/or adolescents/children |
| Population - Exclusion | Nil |
| Design – Included<br>*Search option on trial registers | Interventional |
| Intervention - inclusion | Guided by the WHO definition of a digital health intervention, the current search broadly defines a digital mental health intervention as any component of mental health treatment, prevention, screening and diagnostics, or monitoring that is delivered or supported by digital technologies. This includes but is not limited to web-based programs/activities, mhealth, wearables, virtual or augmented reality systems, chatbots, digital sensing, AI-enhanced algorithms. |
| Intervention - exclusion | Pharmaceutical trials where medication is the primary intervention. |
| Key search terms<br>*Search option on trial registers | Digital OR online OR internet OR smartphone OR mobile OR web OR mhealth OR wearable OR reality or virtual OR augmented OR “immersive technology” OR “VR” OR “ehealth” OR “biofeedback” OR “iCBT” OR “technology-supported” OR “e-mental health” OR sensor |
| Comparator | Any or nil |
| Condition<br>*Primary search option on trial registers | Depression, Anxiety, Psychosis |

| | |
|---|---|
| Outcome - inclusion | Primary outcome is:<br>• Self-reported mental health symptoms (depression, anxiety, psychosis) using a validated assessment measure OR<br>• Clinician-rated mental health symptoms (depression, anxiety, psychosis)<br>OR<br>Mental health diagnosis using validated assessment measure (depression, anxiety, psychosis). |
| Outcome - exclusion | • Inferred mental health status (e.g. through self-selection into a particular activity or group)<br>Reports of self-diagnosis. |
| Data extraction and screening | • Trial registry extracts downloaded and converted to Microsoft Excel workbooks.<br>• Research team reviewed each trial against inclusion criteria using the following screening prompts:<br>Researchers to review TITLES, PRIMARY OUTCOME AND/OR SUMMARY section of trial registry extracts to determine whether trial meets inclusion criteria.<br>• For the title: Does it mention any of the key words related to mental health (depression, anxiety or psychosis)? Does it mention any of the key words related to digital mental health interventions (e.g. web-based, internet, mobile, digital)?<br>• Primary outcome: Does it assess depression, anxiety and/or psychosis? Does it include a validated assessment of symptoms or diagnosis?<br>• Summary: Does it describe the evaluation of digital mental health intervention?<br>Any trials that were deemed unclear or unsure were resolved through consensus meeting. |

## *S2. Instructions for indicator assessment*

| **Supplementary Table S2. Instructions for the indicator assessment** | |
|---|---|
| **Instructions for the non-indicator assessment** | |
| Excel column label | Excel column descriptor |
| Registry | Registry Acronym |
| Trial ID | Trial ID from SPSS |
| Trial Title | Title Reported in SPSS (may be scientific or non-scientific) |
| Trial Type | E.g., RCT or Pilot |
| Start Date | Start date reported in SPSS |
| OECD Income | From SPSS – whether the trial was conducted in high-income country or low income country.<br>0 = Low-to-Middle Income Countries (LMIC)<br>1 = High Income Countries (HIC) |
| Condition | From SPSS – what condition the trial focuses on.<br>1 = Depression<br>2 = Anxiety<br>3 = Psychosis |
| Coder | Initials of coder |
| Protocol (source) | 1 = Protocol Available<br>0 = No protocol available<br>If there is a Protocol available, provide source in brackets e.g., registry, published online.<br>N.B., see process for finding additional sources below |
| Outcomes (DOI) | 1 = Outcomes Publication Available<br>0 = No Outcome Publication Available<br>If there is a publication, provide DOI in brackets. |
| CI contact details | Chief Investigator's contact details as reported on the registry and/or publications. |
| Detail | **Degree of information available:**<br>1 = 1 Output (n.b., all trials will have this i.e., registry)<br>2 = 2 Outputs (e.g., registry + protocol)<br>3 = 3 Outputs (e.g., registry + protocol + outcomes)<br>4 = 4+ Outputs (e.g., registry + protocol + multiple outcomes papers)<br>N.B., for 2-4, the outputs do not need to be those listed e.g., may just be outcomes paper and no protocol. |

| Supplementary Table S2. Instructions for the indicator assessment | |
|---|---|
| **Instructions for the indicator assessment.** | |
| Present | 1= Present in any of the materials (e.g., registry, outcome papers, consent forms, protocols)<br>0= Not present in any of the materials<br>• Key words provided for ctrl+f search functions, however these are not exhaustive, and searches should extend past key words. Add to the key words if a new word that is comping up regularly is found. |
| Sources | **Provide the name of the source, pinpoint location and link to OneDrive.**<br>• E.g. Protocol p 2<br>Sources (such as the trial registry page, publications, protocols, PIS, advertisements) generated as part of the trial are where the information used to assess the criteria are found.<br>To facilitate efficient and transparent interrater reliability checks, these outputs must be downloaded as PDFs, and reuploaded to the OneDrive Folder corresponding to the Trial Registry ID (e.g. NCT5486611).<br>**Steps to link to the OneDrive folder:**<br>1. After the source has been downloaded as a pdf and uploaded to OneDrive – select the three dots next to the source and select 'copy link'.<br>2. In excel – select the cell you have identified the source.<br>3. Either go to 'insert' in the top ribbon, or right click/ctrl+k, and press 'insert link'.<br>4. Paste the link you copied from the OneDrive in the pop-in window and press ok. |
| Source search | **Process for finding registry output:**<br>1. Using the clinical trial ID, find the registry page by searching on the trial registry<br>• Clinical Trials: https://classic.clinicaltrials.gov/ct2/search<br>• WHO: https://trialsearch.who.int/<br>• ISRCTN: https://www.isrctn.com/search?q=<br>• ANZCTR: https://anzctr.org.au/<br>• Pan Africa: https://pactr.samrc.ac.za/<br>2. Save a copy of the trial registry page using the "print screen" function and save as a PDF, and upload to the correct OneDrive folder (nb, some trial registries also provide for downloading as a pdf).<br>**Finding additional outputs (protocols, publications etc.)**<br>1. Check the registry and see if any further sources are provided (included as hyperlinks).<br>2. Search trial ID and CI surname in quotation marks in Google<br>3. Search trial ID in quotation marks in Google. Example: "ISRCTN16378480" AND "Kleinau"<br>4. Copy and paste the trial title in Google Scholar. Check that the author(s) match |

## *S3. Assessment matrix used to measure trial informativeness*

| **Supplementary Table S3. Assessment matrix used to measure trial informativeness** | | | | | |
|---|---|---|---|---|---|
| **Suitability of the team** | | | | | |
| | Indicator | Description | Key words | Example | Rating |
| 1 | Any influence or involvement of industry, proprietary, commercial entities, or the creators of the digital mental health intervention being examined in the research have declared any conflicts of interest [Indicator 2.11] | PRESENT IF there is a reference to conflicts of interests, even if the reference is that there are no conflicts. Does not necessarily need to comprise part of a section on conflicts of interests, may be elsewhere in the text(s). Does not need to reference explicitly 'industry, proprietary, or commercial entities'. These influences may be referenced, among other influences. | Conflict<br>Declar*<br>Competing<br>Interests | "Conflict of interest:<br>T J has consulted for and accepted a research grant from MSD. However, none of the aforementioned has been related to this publication, and MSD has not in any form been involved in planning, analysis, or paper preparation."<br>"Conflict of interests:<br>The authors declare that they have no conflict of interests." | 0<br>Absent<br>1<br>Present |
| **Proposed methodology** | | | | | |
| 2a | Specifies a clear and meaningful primary and secondary outcome(s) and endpoint(s) for the trial | OUTCOMES AND ENDPOINTS-must have both components to be marked present.<br>PRESENT IF primary and/or secondary outcome(s) are provided. Does not need to have secondary outcomes.<br>The outcomes do not have to be overly detailed i.e., they may be on-word e.g., 'depression'. AND PRESENT IF endpoint(s) for the collection of data regarding the primary and/or secondary outcome(s) are provided. End points do not need to be highly detailed, it may simply be at the end of the trial, or at specified timepoints. | Outcome*<br>Primary<br>Secondary<br>Month*<br>Week*<br>Baseline | "Domain: Attitudes toward Homework. Time Point: Baseline, 3- and 6-month post-baseline" | |
| 2b | Provides clear information on how these outcomes will be assessed at the respective endpoints. | PRESENT IF the techniques, scales and/ or tools used for measuring the primary and/or secondary outcomes are provided. Must include sufficient level of detail to ascertain what the tool for measurement is, i.e., provides named scales for assessing outcome measures. | STAI<br>DASS<br>Edinburgh<br>Beck<br>BDI | "The Child and Adolescent Disruptive Behaviour Inventory (CADVI) is a 25-item caregiver-report questionnaire that assess levels of post traumatic distress in youth. Total scores range from 0 to 200 with higher scores reflecting greater symptom severity." | |

| Supplementary Table S3. Assessment matrix used to measure trial informativeness | | | | | |
|---|---|---|---|---|---|
| 3 | Provides a sound justification for the selected digital mental health intervention, the comparators and/or control condition(s) | PRESENT IF there is a description of both the intervention and control groups (if there is a control group). This description allows for ascertaining what both groups will be doing. AND There must be a justification provided for the DMHI. This may be in reference to prior research, theoretical frameworks, or specific needs of the target population that justify the choice. Typically, the intervention will be described in greater detail than the control condition. The details do not need to be matched in order to be marked present, for example, the description of the control may merely mention it was a 'waitlist' control. | Control*<br>Comparator*<br>Justification<br>Rational<br>Validate*<br>Waitlist | "The Vitatalk app uses psychoeducation, cognitive restricting, behavioural activation, gratitude, and practical exercises (such as breathing, relaxation and meditation) to bring about improvement in users' mental health. These techniques and strategies are rooted in CBT and Positive Psychology, two commonly used psychotherapy methodologies that have been used widely in various settings and have registered high effectiveness among patients presenting with various psychological challenges." | |
| 4 | Includes clear instructions and expectations for use of the digital mental health intervention and/or comparators and controls examined in the trial. | PRESENT IF there is mention of instructions given to the participants (DMHI and/or control).<br>May provide the instructions given to the participants but does not need to.<br>May not explicitly state that instructions were given but may mention a process that informed participants of what they will need to do. | Instruct*<br>Expect*<br>Inform*<br>Details<br>Comparator*<br>Control* | "You will receive specific instructions about how to interact with your website"<br>"The control arm of the study will have access to a website with links to mental health resources from WHO and other self-help providers as well as contact information for psychologists and mental health counsellors in Malawi. This website consists of a few pages with basic mental health and coping information, links to mental wellbeing resources, mood meter and standardized mental health tests." | |
| 5 | Specifies how the safety of the digital mental health intervention will be monitored and assessed [Indicator 5.43] and the safety management procedures for participants using or exposed to digital mental health intervention in the trial [Indicator 6.57]. | PRESENT IF safety is one of the primary and/or secondary outcomes.<br>AND/OR<br>PRESENT IF there is a process by which participants will be provided with support throughout and/or after the trial. For example, through access to the primary investigators, or via access to an external support system such as a psychologist. | Safe*<br>Help<br>Risk*<br>Contact*<br>Adverse<br>Distress<br>Support | "This study has made provisions for the procurement of the psychotherapy services of two locally based psychologists, who will be available to provide psychosocial counselling support to participants experiencing distress through the duration of the study. | |

**Supplementary Table S3. Assessment matrix used to measure trial informativeness**

| | | | | | |
|---|---|---|---|---|---|
| 6 | Measures the uptake and engagement with the digital mental health intervention throughout the trial [Indicator 5.40]. | PRESENT IF uptake and/or engagement is one of the primary and/or secondary outcomes(s).<br>AND/OR<br>PRESENT IF uptake and/or engagement is mentioned but not part of primary and/or secondary outcome(s) e.g., through drop-out rates or otherwise. May not explicitly refer to the measurement as 'uptake' or 'engagement', but may refer to other factors of engagement, such as enjoyment or aesthetics. | Uptake<br>Engage* | "Participants in the treatment group will receive daily messages and counseling for the first four weeks of the trial to encourage engagement with Viki, the Vitalk virtual counselor. This will change to every other day during weeks five though eight. | |
| 7 | The data management plan includes protocols to protect data integrity and reduces data loss [Indicator 4.37]. | If a data management plan is mentioned, PRESENT IF the plan explicitly includes data integrity and/or data loss. May not refer to the data management plan as a 'data management plan' but may refer to processes involved in the management of data, such as the software used for storage, blinding procedures and/or anonymisation of data (amongst others). | Data*<br>Data Integrity<br>Data Loss<br>Data<br>Management | "All trial data will be securely stored in the cloud and access to de-identified data restricted to the research team. Access will be controlled by the trial manager for each individual researcher." | |
| 8a | The statistical analysis plan appropriately accounts for missing data. | If there is a statistical analysis plan, PRESENT IF it explicitly mentions measurement to gauge participant engagement. This may include drop-out rates, and specific scales used to measure engagement. | Stat*<br>Measure*<br>Missing<br>Skip<br>Intention to treat (ITT) | "The research team regularly conducted data checks throughout the study to identify missing data problems and prompt necessary actions to resolve them." | |
| 8b | The statistical analysis plan considers the impact of participant engagement in the digital mental health intervention on trial outcomes. | If there is a statistical analysis plan, PRESENT IF it explicitly mentions measurement to gauge participant engagement. This may include drop-out rates, and specific scales used to measure engagement. | Stat*<br>Measure*<br>Engage*<br>Uptake<br>Drop*<br>Attrition<br>Retention | "Given the continuity experience – the proportion of participants completing the pre- and post-treatment assessments for depression, anxiety and stress – of 20-45% reported by Daley, Hungerbuehler et al. (2020), we assume that the dropout rate will be as high as 75% for at least one of the standard mental health assessments. This means that a sample of 512 participants will be required per study arm to yield an effective post intervention sample of 128. Furthermore, if 20% of potential study participants will not meet inclusion criteria or drop out for other reasons, about 640 people will need to be recruited initially per study arm." | |

**Supplementary Table S3. Assessment matrix used to measure trial informativeness**

**Ethical, equitable and open research conduct**

| | | | | | |
|---|---|---|---|---|---|
| 9 | Ensures equitable access and/or usability to the digital mental health intervention for the target population(s) and setting(s), including considerations of technology, language, literacy, and cultural appropriateness for the intended end-users [Indicator 3.13]. | PRESENT IF equitable access and/or is considered. This may include considerations of: technology use, such as ensuring or providing access to the internet (amongst others). considering technology and/ or Language, such as ensuring that the materials are provided in a certain language relevant to the end-users and/or literacy, such as ensuring that that the materials are legible for the intended end-users, e.g., if the intervention is targeted for children, including age-appropriate wording and/or cultural appropriateness, such as ensuring the intervention is piloted in the intended end-users' cultures. Usability can include any element of trial/product design that aims to make the DMHI easier to use. | Equit*<br>Usable<br>Usability<br>Access*<br>Language<br>Literacy<br>Cultur* | "While the Vitatalk app was previously adapted to the Malawaian context, the FGDs will further explore its cultural appropriateness. A mix of in-person and anonymous virtual FGDs are planned given the sensitive nature of mental health."<br>"Participants will receive an allotment of 10Gb data bundle allowance... with a selected mobile carrier to eliminate any barriers of accessing the internet during the trial. Those not using the preferred provider at the time of the trial will receive a free SIM card.'<br>"Interviewees will also be asked to complete the System Usability Scale via REDCap survey to provide quantitative data on Adhere.ly's usability." | |
| 10 | Addresses ethical issues related to emerging technologies and data collection within digital mental health intervention [Indicator 3.14] including privacy, data security, confidentiality, and adherence to relevant local regulations [Indicator 3.15]. | PRESENT IF ethical issues related to the DMHI are raised including privacy and/ or data security and/or confidentiality and/ or regulations.Mark present if the trial includes details on privacy, data security, confidentiality. AND/OR Mark present if the trial references adherence/compliance to local laws/ regulations or international standards. | Ethic*<br>Privacy<br>Data Sec*<br>Confidential*<br>Regulation*<br>Legal*<br>Law | "All data collected during the trial will be securely stored in a REDCap database... only IRB-approved and trained study personnel will have access to the REDCap project and access will be limited to information and modules that are required for them to complete their assigned study-related tasks. All identifiers will be marked as such in REDCAp and will not be included in the final exported dataset... we will use the following security measures to protect data sources... (3) computers and servers containing data will be password-protected to prohibit unauthorised access... (5) Adhere.ly includes state-of-the-art technical infrastructure-including encryption and other software, security practices, and business operational practices to ensure compliance with all major governing legislation, including HIPAAA.." | |
| 11 | Ensures that the use of digital consent processes effectively meets the ethical standards for informed consent [Indicator 6.52]. | PRESENT IF the trial identifies the ethical standards that have been followed and/ or mentions that informed consent will be obtained. It is unlikely that many of the trials will explicitly state the ethical guidelines that have been adhered to, so the bar for inclusion is quite low i.e., mentions informed consent is obtained. | APA<br>Ethic*<br>Consent | "The study will be conducted with full adherence to ethical standards as expressed in the APA Code of Ethics on Psychological research, as well as the Declaration of Helsinki. Before commencement of the study, relevant authorization will be sought from University of Malawi Research Ethics Committee (UNIMAREC). Participation in the study will be voluntary and participants will be informed of this, and that they may withdraw at any time. Participants who agree t participate in the study will be informed clearly what the study is about and their information will be used. Informed consent will be obtained online upon registration at the study web-portal." | |

| Supplementary Table S3. Assessment matrix used to measure trial informativeness | | | | | |
|---|---|---|---|---|---|
| 12 | Ensures any data monitoring procedures and follow-up actions are made clear to participants as part of informed consent [Indicator 6.58]. | PRESENT IF the trial mentions data monitoring and/or follow-up actions specifically in the context of informed consent. This may be ascertainable if the informed consent form is provided and this is clearly visible. AND/OR If an output other than the informed consent form specifically states that data monitoring procedures are included in informed consent. | Monitoring<br>Follow-up<br>Follow up<br>Consent | "Data analysis will begin after the final workshop is concluded. There will be no interim sharing or analysis of trial data other than monitoring sample size in the two treatment arms and data completeness and appropriateness per data analysis plan for the entire trial data set. Data monitoring will be done by the trial manager [and communicated to participants in the consent form]. | |
| 13 | Monitors deviations to protocol and ensures the effects of these deviations will be managed [Indicator 5.44]. | PRESENT IF deviations to the protocol are identified and outlines an approach for managing those deviations. Deviations include any departure from the study protocol. This includes both researcher and participant deviations. | Changes<br>Deviations<br>Depart*<br>Protocol | "Changes to the protocol Due to the COVID-19 pandemic, three main changes to the protocol were required. First, the original primary outcome measure, a real-world behavioural assessment task (O-BAT),11 had to be replaced part way through the trial. Due to the COVID-19 lockdown measures implemented in March, 2020, we were not allowed to continue to administer the O-BAT." | |
| 14 | Ensures timely and comprehensive reporting of results and outputs that will be accessible to stakeholders (for example, end-users, policy makers, communities, industry, clinicians/practitioners, service providers) [Criteria 5]. | PRESENT IF there is a clear reference to how the results will be disseminated/shared to external stakeholders. This includes but is not limited to research outputs such as publications, conference papers, guidelines, frameworks, websites etc. | Results<br>Publication<br>Policy<br>Conference<br>Dissemination | "The results of this study will be published in an international, peer-reviewed journal and a copy will be submitted to UNIMAREX. These results can also be presented at relevant research conferences, whether local or international. The results of this research will also be disseminated through presentations in fora that will inform policy at a national level and health system strengthening approaches supported by development organizations such as the Ministry of Health, health facilities from which participants were recruited, USAID, Chemonics and University Research Co( URC), and academic institutions such as the University of Malawi." | |
| **Potential and Impact** | | | | | |
| 15 | Provides a viable plan for the dissemination and translation of the digital mental health intervention post-trial, if found to be effective | PRESENT IF there is a reference to the application of the digital mental health intervention post-trial. This can be any reference to translating/implementation of the digital mental health intervention into clinical practise or commercialisation as a product. | Translat*<br>Practic*<br>Clinical | "Researchers and the general scientific community will benefit from the knowledge gained from the study. This includes knowledge about how to design and implement a user-centred and stakeholder-informed program that supports patient and provider adherence to evidence-based practice as well as effective vs less effective strategies for implementing health technology solutions that promote this adherence in mental health practice settings in the community." | |

## *S4. Examples of indicators from the highly informative trials*

<table>
<tr><th colspan="3">Supplementary Table S4. Example of met indicators extracted from trials with perfect informativeness scores (n=8).</th></tr>
<tr><th>Indicator</th><th colspan="2">Example Extract from Trial Output</th></tr>
<tr><td>Any influence or involvement of industry, proprietary, commercial entities, or the creators of the DMHI being examined in the research have declared any conflicts of interest.</td><td colspan="2">“Conflicts of Interest:<br><br>The current study was funded by juli Health. AK, BD, BH, JS and JFH are shareholders in juli Health. AK has received consultancy fees from juli Health and Wellcome Trust. BD, BH, JS and JFH are a co-founders of juli Health. JFH has received consultancy fees from juli Health and Wellcome Trust. KE and MM have no conflicts of interest. The funders played no part in the analysis of the data.”</td></tr>
<tr><td rowspan="7">Specifies a clear and meaningful primary and secondary outcome(s) and endpoint(s) for the trial</td><td colspan="2">Primary Outcome Measures</td></tr>
<tr><td>Outcome Measure</td><td>Timeframe</td></tr>
<tr><td>Level of auditory hallucinations</td><td>12 Weeks from inclusion</td></tr>
<tr><td colspan="2">Secondary Outcome Measures</td></tr>
<tr><td>Outcome Measure</td><td>Timeframe</td></tr>
<tr><td>Frequency of auditory hallucinations</td><td>12 Weeks from inclusion</td></tr>
<tr><td colspan="2"></td></tr>
<tr><td rowspan="6">Provides clear information on how these outcomes will be assessed at the respective endpoints.</td><td colspan="2">Primary Outcome Measures</td></tr>
<tr><td>Outcome Measure</td><td>Measure Description</td></tr>
<tr><td>Level of auditory hallucinations</td><td>Level of auditory hallucinations measured with the Psychotic Symptoms Ratings Scales (PSYRATS-AH) total score at cessation of treatment at 12-weeks (score on PSYRATS-AH: 0-44. A score of 0 is the minimum score and 44 is the maximum score i.e. a score of 44 is the worst possible outcome.</td></tr>
<tr><td colspan="2">Secondary Outcome Measures</td></tr>
<tr><td>Outcome Measure</td><td>Measure Description</td></tr>
<tr><td>Frequency of auditory hallucinations</td><td>PSYRATS-AH-Frequency</td></tr>
<tr><td>Provides a sound justification for the selected DMHI, the comparators and/or control condition(s)</td><td colspan="2">“Video gaming is a promising intervention for cognitive and social impairment in patients with schizophrenia. A number of gaming interventions have been evaluated in small-scale studies with various patient groups, but studies on patients with schizophrenia remain scarce and rarely include the evaluation of both clinical and neurocognitive outcomes. In this study, we will test the effectiveness of two interventions with gaming elements to improve cognitive and clinical outcomes among persons with schizophrenia.”</td></tr>
<tr><td>Includes clear instructions and expectations for use of the DMHI and/or comparators and controls examined in the trial.</td><td colspan="2">“During the exercise, the VTA will provide support with instructions and images of the steps to be carried out.”</td></tr>
<tr><td>Specifies how the safety of the DMHI will be monitored and assessed and the safety management procedures for participants using or exposed to DMHI in the trial.</td><td colspan="2">“Safety of participants will be supported in two ways. First, both versions of the app contain crisis support details that can be used at any time. Second, participants who report distress to a telephone facilitator will be directed by that facilitator to further support as appropriate (eg, a general practitioner or local emergency department). A report of distress will not be considered ground for immediate modification or cessation of participation in the trial, but participants are able to choose to withdraw from the study at any time. Study staff will continually monitor for and record adverse events when or if they arise.”</td></tr>
</table>

| Supplementary Table S4. Example of met indicators extracted from trials with perfect informativeness scores (n=8). | |
|---|---|
| **Indicator** | **Example Extract from Trial Output** |
| Measures the uptake and engagement with the DMHI throughout the trial. | "In the intervention group, approximately 80% engaged in the full therapy course with seven sessions." |
| The data management plan includes protocols to protect data integrity and reduces data loss. | "Only authorized research personnel will have access to the password protected electronic database. No unauthorized access will be possible. A separate list linking codes with names will be kept in a secure place. The data will be introduced and analysed by computers. As for Internet use and monitoring by means of mobile apps, data protection systems will be designed (using secure passwords, encryption, etc.). The researchers will have access to the database using a password. Also, in order to protect all<br>information, we will follow the AES (Advanced Encryption Standard) strategies for personal password use and data encryption. The study researchers will promise to not reveal data from which personal and<br>health information about the participants could be deduced. The same principles will be taken into consideration in the dissemination of data in the publication of scientific papers and the presentation of research reports at scientific conferences." |
| The statistical analysis plan appropriately accounts for missing data. | "Methods in analysis to handle protocol non-adherence and any statistical methods to handle missing data {20c}<br>Missing data are handled implicitly by the linear mixed models through full information maximum likelihood. Analyses will be conducted according to the intention-to-treat principles, i.e. analysing individuals to their allocated groups regardless of, e.g., protocol non-adherence." |
| The statistical analysis plan considers the impact of participant engagement in the DMHI on trial outcomes. | "7.2 Attrition<br>The number and percentage of losses to follow up among participants will be reported for the two trial arms, and the reasons will be recorded. All deaths of the participants will be reported separately.<br>We will compare the attrition rate by assigned treatment status to test whether the probability of attrition differs by treatment type. We will analyse:<br>$\gamma i=Treatment*\beta 1+Xi\lambda+\varepsilon 1$<br>'i' indexes individuals, $\gamma i$ is an indicator variable for whether the participant attrited from the round, Treatment I is an indicator variable for a person assigned to the treatment and $Xi$ is a vector of prespecified covariates, including individual and household characteristics (age, sex, marital status, highest educational level, household asset index, and food security) and mental health outcomes at baseline. We will display this analysis for each round of data collection." |

| Supplementary Table S4. Example of met indicators extracted from trials with perfect informativeness scores (n=8). | |
|---|---|
| **Indicator** | **Example Extract from Trial Output** |
| Ensures equitable access and/or usability to the DMHI for the target population(s) and setting(s), including considerations of technology, language, literacy, and cultural appropriateness for the intended end-users. | "Study setting<br>The study will be based in the Bushbuckridge subdistrict of Mpumalanga province, South Africa. While South Africa is classified as a middle- income country, large inequalities exist, and the study setting represents a rural area characterised by socioeconomic disadvantage and high rates of youth unemployment. […]<br>Patient and public involvement<br>Adolescents and members of the public have been involved at several stages of the study. The intervention was developed through extensive formative research and user- centred design with adolescents in the study area. We also conducted participatory workshops with adolescents and met with local educators, healthcare workers and relevant non- governmental organisations to obtain their input on trial methods, including recruitment and risk management strategies. We will seek the involvement of adolescents and members of the public in developing appropriate methods to disseminate study findings. […]<br>Intervention<br>All participants in the intervention and control arms will be given the entry- level Samsung Galaxy A2 Core Android smartphone, which they can keep at the end of the study. Furthermore, participants in both groups will receive 200 MB of mobile internet data at six different time points (0, 2.5, 5, 7.5, 11 and 24 weeks) to ensure they have data to use the app and complete the online surveys." |
| Addresses ethical issues related to emerging technologies and data collection within DMHIs including privacy, data security, confidentiality, and adherence to relevant local regulations. | "Data will be kept confidential by deidentifying it and utilising a study ID for each participant. Identifying participant information will be password protected and stored separately from the rest of their data. The study will be run centrally from Phoenix Australia, so only Phoenix Australia researchers will have access to the file containing identifiable information. Survey data will be collected via REDCap which is managed by Phoenix Australia and stored on Phoenix Australia, University of Melbourne computers using a VPN (Virtual Private Network) and secured by password access. Meta- data and data analytics from the study app will be stored on the Cloud Firestore database, which allows data to be stored securely on the cloud in Google's Australian data centre. From there, app data will be downloaded on demand using a purpose- built admin portal that only Phoenix Australia researchers named on this application have access to." |
| Ensures that the use of digital consent processes effectively meets the ethical standards for informed consent. | "Informed consent to participate in the trial will be obtained electronically from all participants." |
| Ensures any data monitoring procedures and follow-up actions are made clear to participants as part of informed consent. | "How will we use information about you?<br>We will need to use information from you for this research project. This information will include your name, contact details (email, mobile number). We will use this information to do the research or to check your records to make sure that the research is being done properly. We will keep all information about you safe and secure. Once we have finished the study, we will keep some of the data so we can check the results." |
| Monitors deviations to protocol and ensures the effects of these deviations will be managed. | "In response to the COVID-19 pandemic, we anticipated heightened stress in this group. Therefore, the study design was modified to allow all eligible candidates to receive the app immediately for download after registering for the study. We also minimized the number of reminder messages to only 1 message to avoid adding to participants' stress by requesting their continued study participation. By avoiding these "push factors," the study became more naturalistic and consequently had greater ecological validity than originally planned." |

| Supplementary Table S4. Example of met indicators extracted from trials with perfect informativeness scores (n=8). | |
|---|---|
| **Indicator** | **Example Extract from Trial Output** |
| Ensures timely and comprehensive reporting of results and outputs that will be accessible to stakeholders (for example, end-users, policy makers, communities, industry, clinicians/ practitioners, service providers). | “Dissemination plans<br>The results of the study will be disseminated to the scientific audience, the general public and trial participants. In addition to journal publications and conference presentations, the results will be discussed with relevant patient and clinical interest groups.” |
| Provides a viable plan for the dissemination and translation of the DMHI post-trial, if found to be effective. | “The goal of this phase of the project is to understand the ways in which SOLAR, traditionally a programme delivered face to face by trained, non- specialist providers (ie, ‘coaches’), can be translated into a self- guided digital health programme, as well adapted to meet the specific needs and preferences of emergency service workers.” |

## *S5. Inter-rater reliability scores*

| **Supplementary Table S5. Inter-rater reliability scores calculated throughout the review process** | |
|---|---|
| **Indicator** | **Fleiss' Kappa (k) N = 152** |
| Any influence or involvement of industry, proprietary, commercial entities, or the creators of the digital mental health intervention being examined in the research have declared any conflicts of interest. | .959 |
| Specifies a clear and meaningful primary and secondary outcome(s) and endpoint(s) for the trial | .663 |
| Provides clear information on how these outcomes will be assessed at the respective endpoints. | .269 |
| Provides a sound justification for the selected digital mental health intervention, the comparators and/or control condition(s) | .592 |
| Includes clear instructions and expectations for use of the digital mental health intervention and/or comparators and controls examined in the trial. | .433 |
| Specifies how the safety of the digital mental health intervention will be monitored and assessed and the safety management procedures for participants using or exposed to digital mental health intervention in the trial. | .676 |
| Measures the uptake and engagement with the digital mental health intervention throughout the trial. | .596 |
| The data management plan includes protocols to protect data integrity and reduces data loss. | .455 |
| The statistical analysis plan appropriately accounts for missing data. | .859 |
| The statistical analysis plan considers the impact of participant engagement in the DMHI on trial outcomes. | .585 |
| Ensures equitable access and/or usability to the digital mental health intervention for the target population(s) and setting(s), including considerations of technology, language, literacy, and cultural appropriateness for the intended end-users. | .537 |
| Addresses ethical issues related to emerging technologies and data collection within digital mental health interventions including privacy, data security, confidentiality, and adherence to relevant local regulations. | .667 |
| Ensures that the use of digital consent processes effectively meets the ethical standards for informed consent. | .794 |
| Ensures any data monitoring procedures and follow-up actions are made clear to participants as part of informed consent. | .434 |
| Monitors deviations to protocol and ensures the effects of these deviations will be managed. | .629 |
| Ensures timely and comprehensive reporting of results and outputs that will be accessible to stakeholders (for example, end-users, policy makers, communities, industry, clinicians/practitioners, service providers). | .559 |
| Provides a viable plan for the dissemination and translation of the digital mental health intervention post-trial, if found to be effective. | .658 |
